# Mixing Sows into Alternative Lactation Housing Affects Sow Aggression at Mixing, Future Reproduction and Piglet Injury, with Marked Differences between Multisuckle and Sow Separation Systems

**DOI:** 10.3390/ani9090658

**Published:** 2019-09-05

**Authors:** Emma C. Greenwood, Jonathon van Dissel, Jessica Rayner, Paul E. Hughes, William H. E. J. van Wettere

**Affiliations:** 1 School of Animal and Veterinary Sciences, The University of Adelaide, Roseworthy 5371, Australia (J.v.D.) (W.H.E.J.v.W.); 2 South Australian Research and Development Institute (SARDI), Roseworthy 5371, Australia; 3 Paul Hughes Consulting, North Adelaide 5006, Australia

**Keywords:** aggression, lactation housing, piglet, production, stress, sow

## Abstract

**Simple Summary:**

Alternative lactation housing could reduce aggression when sows are mixed. We aimed to compare the effects of mixing sows during lactation (with or without piglets) at weaning and after insemination and determine the effects of mixing strategies and lactation housing on the piglet. Sows in the multisuckle treatment were the least aggressive, had the fewest injuries around mixing, and gave birth to the most piglets at the subsequent litter, with multisuckle housing having no apparent ill effects on the piglets. There was greater aggression in sows separated from their piglets for seven hours daily in lactation (SEP) than any other method. Multisuckle housing appears to be an effective way of decreasing aggression at the point of mixing, whilst optimizing sow reproduction. The behavioural response to mixing was similar when it occurred at weaning or after insemination, with the highest incidence of aggression observed in sows mixed without their piglets during lactation.

**Abstract:**

Alternative lactation housing could reduce aggression when sows are mixed. We aimed to compare the effects of mixing sows in lactation (with or without piglets), at weaning or after insemination, and determine the effects of lactation housing on the piglet. This study used 120 multiparous Large White × Landrace sows and 54 focal litters. The sows were mixed into groups of six and allocated to multisuckle from day 21 lactation (MS), separated from litter and housed in groups, with piglets left in the crate for seven hours daily from day 21 lactation (SEP), mixed at weaning (day 28 lactation) (WEAN) and mixed after artificial insemination (AI) (MAI; 4 ± 1 day after last AI). Behaviour, saliva for free salivary cortisol concentration and injury counts were taken on M-1 (before mixing), M0 (mixing), M1 and M6. Piglets were weighed, injury-scored and bloods taken for cortisol. There was reduced aggression, seen as fights, bites and knocks in MS compared to the other treatments on all days (*p* < 0.05). MS sows had no fights on M1 and M6 and had more piglets born in the subsequent farrowing. Piglet weight, cortisol and mortality were unaffected by treatment (*p* > 0.05). MS piglets had greater injury scores immediately after moving to multisuckle and lower injuries around weaning (*p* > 0.001). Multisuckle housing could decrease aggression and stress at mixing in sows, with changes in the time of peak piglet injury (at mixing rather than at weaning) but overall no negative effects on the piglets.

## 1. Introduction

In Australia, the majority of breeding sows are housed in farrowing crates prior to and during parturition, where they remain until their piglets are weaned [1]. Once weaning is complete, there are two main management strategies employed; either the sows are mixed immediately into groups, or they are initially housed individually and then mixed during the first week after insemination. Which management technique a farm employs is primarily dependent on the flow of the farm and the available infrastructure. From a welfare perspective, the second system described is not ideal. Confining the sow after weaning restricts its ability to exhibit certain behaviours, such as roaming and socialisation [2,3] and may increase restlessness and irritability [4,5]. Abrupt weaning inflicts social, environmental and nutritional stresses on the piglet, which can lead to growth retardation, decreasing production efficiency [3,6]. Mixing sows into groups commonly results in high levels of aggression [7,8,9], with the subsequent fighting and stress impairing reproduction [10,11,12]. Stress during weeks 2–4 of gestation can impair embryo attachment and maternal recognition of pregnancy, as well as reducing farrowing rates and litter sizes [10,13,14,15,16]. Stress during the follicular phase affects the timing and duration of oestrus, the luteinizing hormone surge, the process of ovulation and the pregnancy rate [17].

Prior to 2015, few studies had examined how mixing sows in early pregnancy, or before insemination, affects behaviour [8]. Recently, the area has become one of increased interest, focusing on describing the effect on behaviour and subsequent reproduction in the sow and piglet production also. Modifying housing during late lactation may improve sow welfare by enabling natural behaviours (such as sow-regulated lactation and social interaction). However, sow and litter productivity must be improved, or at least maintained, compared to conventional systems. Housing several sows and their litters together in groups (multi-suckling), or temporarily separating sows and litters during lactation, may benefit piglets by ensuring a more gradual weaning [5]. It may also result in lactation ovulation, improving reproductive efficiency [5,15]. Multi-suckle housing, or temporary mixing of sows during lactation, might reduce the aggression and stress inherent with the formation of new hierarchies. In rats and sheep, the neuroendocrine response to stress is attenuated during lactation [18,19,20], suggesting that stress and the behavioural response to group formation might be lower in lactating sows compared with post-weaning sows. The current study was designed to compare the sow’s response to the grouping/mixing event (in terms of behaviour, stress and reproduction), when mixed at different times and in the presence or absence of piglets. The four treatments were chosen to mirror the two main management strategies in production systems (mixing at weaning and after insemination, which have not been compared to each other extensively) and then two novel mixing strategies of mixing in lactation, with and without the presence of piglets. The second aim was to determine the effect of lactation housing on piglet growth, injury and survival around the time of weaning.

## 2. Materials and Methods

### 2.1. Animals and Treatments

This pilot study was conducted in accordance with the guidelines set out in the ‘National Health and Medical Research Council (NHMRC)/Commonwealth Scientific and Industrial Research Organization (CSIRO)/Australian Animal Commission Australian Code of Practice for the Care and Use of Animals for Scientific Purposes [21] and with the approval of The University of Adelaide Animal Ethics Committee (Animal Ethics Committee Approval Number: S-2014-161A). All animal work was carried out at The University of Adelaide piggery, Roseworthy, South Australia.

The pilot study utilized 120 multiparous (parity 1–7) Large White × Landrace sows, and was conducted over five replicates, from April to September (Autumn to Spring). Sows were selected from one breeding batch per replicate and each replicate had all four treatments administered. Focal litters from 54 sows were selected for piglet measures. The 54 focal litters (for measurements taken from the piglets) came from 18 MS, 18 SEP, 9 WEAN and 9 MAI sows (since the WEAN and MAI treatment were identical for the piglets (differing only in the sows’ treatment), this is discussed as control, C, in the piglet sections). Sows were mixed into groups of six and sorted based on achieving an even parity mix across treatments (average parity 3.7 ± 0.8), thereby preventing any confounding effects of parity on sow behaviour and reproductive performance. All sows and litters were in identical lactation crates before treatments were imposed.

**Figure 1:**
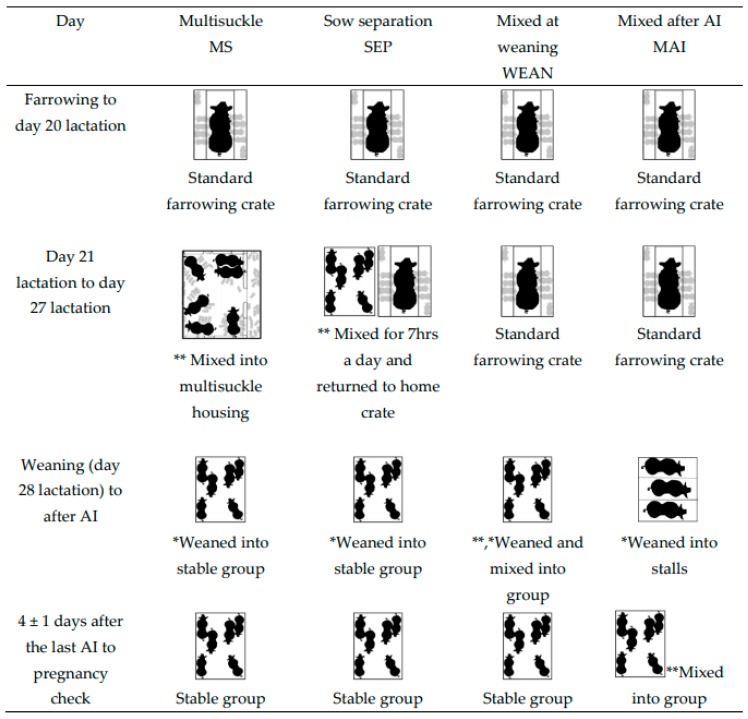
Lactation housing treatments imposed onto sow and piglets ^1,^* Highlights the timing of weaning stressor, ** highlights the timing of mixing ^1^. At weaning (*), all piglets were weaned into one weaner accommodation room.

**Figure 2:**
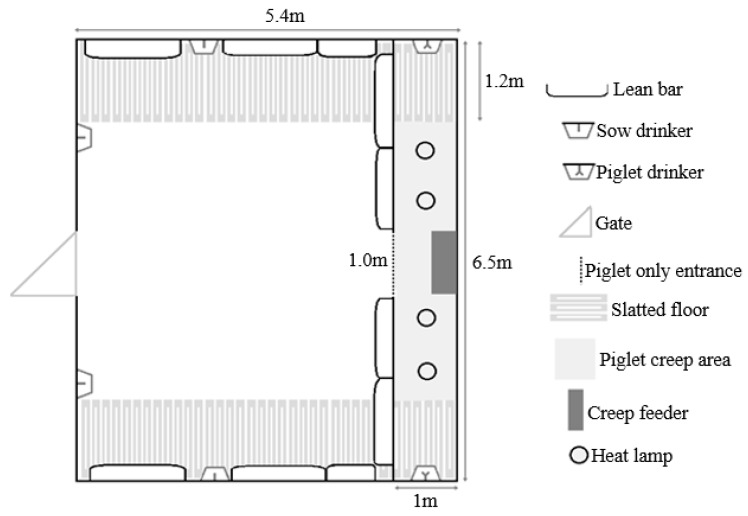
Diagram of the layout of the multisuckle treatment (MS) housing (with 6 sows and litters per group).

There were four treatment groups (see Figure 1 for diagrammatic view of mixing and weaning events). Figure 2 demonstrates the layout of housing for mixing treatments.

1. Group-housed sows and litters (multisuckle) from Day 21 lactation (MS).
  MS sows (*n* = 30) were mixed with their litters into groups of 6 (allowing 4.7 m^2^ of sow accessible space plus 1.1 m^2^ of creep per litter, or 5.8 m^2^/sow and litter total area, Figure 2) beginning on Day 21 post-farrowing (which was calculated from the piggery’s set weaning day, which was an average day 28 of lactation). The sows and litter remained in the same housing until weaning. Piglets were then weaned from the sows on day 28 of lactation into the same stable groups and sows were inseminated in the same groups (2 m^2^/sow after weaning).
2. Sows separated from crates and litters for seven hours each day (by mixing the sows alone into a group pen, and later returning to their home crates and litters) (SEP). Beginning on Day 21 post-farrowing, SEP sows (*n* = 30) were removed from their standard lactation crate and mixed away from their piglets, into groups of six, for seven hours daily (from 06:50 to 13:50). Piglets remained in the home crate and sows were returned to the piglets after the days mixing. Sows in the SEP treatment group had their piglets weaned (lactation day 28) from them and were then mixed into, and inseminated in, these same stable groups (2 m^2^/sow after weaning).
3. Sows mixed together after weaning at Day 28 lactation (WEAN). WEAN sows (*n* = 30) were weaned from standard farrowing crates directly into pens of 6, providing 2 m^2^/sow, and inseminated in the same stable groups.
4. Sows placed into stalls and then mixed 4 ± 1 days after the last insemination (MAI). MAI sows (*n* = 30) were weaned into stalls on Day 28 lactation. After their last AI (4 ± 1 days), the sows were mixed in groups of six (2 m^2^/sow).

Measurements for all groups were taken in a 7-day block around mixing, on Days M-1 (day before mixing), M0 (mixing), M1 and M6. At weaning (27.04 ± 2.05 days after farrowing), all the sows were housed in identical pens, allowing 2 m^2^/sow, constructed with half-concrete/half-slatted flooring, apart from MS sows during lactation (Figure 1).

All sows were exposed to boars, with 20 min of fence-line exposure daily across all treatments. This was done in order to stimulate the onset of oestrus and continue the production flow. The boar was moved to see the sows in MS and MAI (in stalls) and sows moved to the boar in SEP and WEAN. The methodology of exposure was different across treatments as we implemented the least labour-intensive method for each treatment, as would be chosen if implementing them in commercial settings. Exposure began at around 07:30, until exhibition of oestrus, and then sows received three artificial inseminations 24 h apart (if still standing for the third). Boar exposure for each group began after sows were moved into groups or upon being weaned (whichever came first), dependent on their treatments. Therefore, the MS and SEP groups were exposed from the day after the commencement of treatment (Day 22 of lactation), and exposure was performed daily until AI was complete. The WEAN and MAI groups were exposed from the day after weaning until AI was complete. After pregnancy confirmation, on Day 25 of gestation via ultrasound, all pregnant animals were relocated to a single straw-based shelter (at approximately 4.2 m^2^/sow), in a group of up to 40, where they remained until farrowing.

### 2.2. Feeding

Feeding was standardised so that feeding amount was the same across treatments within all stages of the reproductive cycle. In lactation, sows were each fed 8 kg of lactation diet per day (18.7% protein, 4.5% crude fibre, 1.2% total lysine, 14.2 MJ/kg DE), over two feed drops, one at 07:30 and the second at 16:00. The second drop was delivered an hour or more after all measurements had been taken so that anticipation of feed did not affect sampling. The SEP sows were fed their first drop upon being moved into their separation group and their second drop in the afternoon, after being returned to their family crate. After weaning, sows were fed up to 6 kg of standard dry sow diet (13.8% protein, 5.0% crude fibre, 0.7% lysine, 13.0 MJ/kg DE) once daily at 07:30 until five days after weaning. After this, they were fed the same dry sow diet at 2.5 kg per sow. Once mixed into their groups and up to Day 28 of gestation, the sows were manually floor-fed at the front of the pen. Water was available ad libitum via nipple drinkers located within all crates, stalls and pens. The SEP sows were fed in groups for the 07:30 feed, after separation from the litters, and in the crates for their 16:00 feed, after returning to their litters.

### 2.3. Piglets

Creep nutrition was provided ad libitum to all piglets (21% protein, 0.085 average Lysine, 15.5 MJ/kg Digestible Energy (DE)). Piglets had constant easy access to nipple drinkers, dispensing fresh water in all housing systems. At weaning (Lac 28/W0) all piglets were mixed into a weaner room, which contained all piglets from all treatments. The piglets were kept in this room with ad libitum water and creep feed until three days after weaning (when they were returned to the piggery). This allowed for weighing of the piglets after weaning, without interfering with the larger group of piggery weaner piglets.

### 2.4. Sow Behavioural Observations

Cameras were fixed above each group pen, allowing observation of the entire pen (Camera: Legria HFR26, Canon, Sydney, Australia). Video was recorded between 07:00 to 13:00 on M0, M1, M6. Sow behaviour was recorded for six hours each day, capturing mixing and movement from lactation housing to gestation housing (after weaning) on the relevant days and all morning feeding events. Behaviour was observed and analysed continuously for the recorded 6 h and as described in Greenwood et al. (2016) [9]. In summary, behavior was observed using continuous sampling. Behaviour was analyzed either as a continuous variable, in that all sows had to be performing one of the defined behaviors at any given time during the recording, or a point behavior, which did not have a duration, such as a knock. There were some additional behaviours added to the ethogram to account for the MS housing (sow and piglet interactions, Table 1). To allow for the differences in behaviour coded for each treatment (i.e., MS sows could suckle litters, but no other treatment could), an active state which has been analysed for discussion is defined as the total recording length minus the time spent resting (total time spent active, see ethogram, Table 1). When boar exposure occurred, the code ‘boar exposure’ was used (Table 1) and no other behaviour was recorded during this time. Given that some pens did not receive boar exposure on certain days, behaviours were calculated as a percentage of the observation time minus the time taken up by boar exposure.

### 2.5. Sow Injury Counts and Locomotion

Skin injury counts were recorded at 14:00 on M-1, M0, M1, M6. A modification of the assessment described by Karlen et al. (2007) [22] was used to describe injury counts, with methodology for injury counts to be found in Greenwood et al. (2016) [9]. To summarize, each side of the sow’s body was divided into 21 areas and the number of skin injuries in each area was counted and then summed. Injuries were classified as a scratch, abrasion on skin or crack on hoof, open cut on skin or broken hoof, old cut or scar, or abscess. Injury was also analysed as total injuries, injuries on the front (shoulder forwards) and back (behind shoulder) of the sow and fresh/new injuries (obviously new, weepy or bloody). Locomotion scores were also recorded. A score of 0 was given when a sow’s ability to stand was unaffected and all legs bore weight evenly; 1 when the sow was not considered to be lame, but movement was compromised; 2 when the sow was moderately lame, and the ability to stand was obviously reduced and; 3 when the sow was severely lame and the ability to stand and move was severely restricted. Only 2 sows received a score of 1 (both from the SEP group) and the remainder scored 0. Thus, there was insufficient data for analysis.

### 2.6. Sow Saliva Sample Collection and Analysis

Saliva samples were collected from all sows, on M-1, M0, M1, M6 using cotton plugs (Salivettes, Sarstedt Australia, SA, Australia) attached to plastic ties. Each sow was allowed to chew on the salivette for a maximum of two minutes to obtain the sample. When it was not possible to obtain the sample in the two-minute time period, the sow was left and no sample was obtained for this animal. This failure to obtain a saliva sample occurred five times during the study period. Sampling began at 13:30 on each sample day and concluded approximately one hour later. This sampling method was chosen due to it being a low stress methodology, with very little manipulation of the animals needed. Samples were then centrifuged at 2012× *g* for 10 min at room temperature and stored at −20 °C until analysis. The samples were sent to the School of Animal Biotechnology, University of Western Australia for analysis of salivary cortisol using a MP Biomedicals I125 RIA Cortisol Kit (# 07-221106) (MP Biomedicals Australia, Seven Hills, NSW, Australia). The limit of detection was 1.5 ng/mL and the mean intra-and inter-assay coefficients of variation were 5.2 and 9.1%, respectively.

### 2.7. Sow Reproduction

The pregnancy rate (the number of sows pregnant when measured by ultrasonography at approximately Day 25 after mating), the farrowing rate (measured by the number of sows that farrowed at full term following mating) and the total litter size were recorded. First, AI and weaning date were used to calculate the average number of days to standing heat, the incidence of lactational oestrus and the number of sows expressing oestrus behaviour before, and after, weaning. Lactation oestrus was determined as sows expressing oestrus behaviour before weaning on Day 28 of lactation.

### 2.8. Piglet Measures

All piglets were weighed individually and injury-scored on Day 20 lactation (Lac 20), Day 24 lactation (Lac 24), Day 28 lactation/ weaning (Lac 28/W) and 1 day (W1) and 2 days (W2) after weaning. For the injury score, a score of 0–3 was given, using a scaling system adapted from Widowski et al. (2003) [23]. A score of 0 was given if no scratches or areas of skin loss were evident on the animal. A score of 1 (mild) was given if one to three small (<2 cm) scratches or areas of abraded skin is evident, or scratches were present on the face or back only. A score of 2 (moderate) was given if one to three larger (>2 cm) scratches, or areas of abraded skin were observed on the back and/or face. A score of 3 denotes more than three scratches (usually >2 cm) or where there are larger areas of superficial skin loss and scratches appear on both the face and back.

Jugular venepuncture (via a BD (Becton, Dickinson and Company) precision glide needle 21 g, 1 inch, BD, North Ryde, NSW, Australia and BD Vacutainer Lithium Heparin Blood Collection Tubes, 5 mL, BD, North Ryde, NSW, Australia) was used to collect blood samples from four focal piglets in each litter (same piglets on each measurement day, based on a power calculation which suggested that 72 piglets per treatment would be adequate to see cortisol differences), two days prior to treatment start (Lac 19), on the fourth day of treatment (Lac 24) and the day after weaning (W1). Piglets were bled for the cortisol measure (unlike sows which had saliva collected) as we were concerned that the young piglets would not give enough saliva easily (after trialing in several 3–4-week-old piglets before the trial began). Piglet baseline measures for cortisol were taken on Lac 19 (rather than Lac 20), as this ensured that the baseline measures from the piglets did not interfere with the sows measures on Lac 20 (the day before lactation housing was imposed), and vice versa. Blood samples were stored on ice until centrifugation at 500× *g* for 10 min, after which plasma was removed and stored at −20 °C until analysis. Plasma concentrations of cortisol were measured in duplicate by radioimmunoassay using Immuchem^TM^ Coated Tube Cortisol^125^ RIA kit (MP Biomedicals, Santa Ana, CA, USA). The limit of detection was 3.4 ng/mL. Quality control samples (9.38, 33.6 and 147.3 mg/mL) were used to estimate the intra-assay (3.9%, 4.5% and 7.0%) coefficients of variation.

### 2.9. Statistical Analyses

Before analysis, data were checked for normality by examining the distribution of residual plots. This resulted in the majority of the data being transformed, as the statistical package which has been used assumes normality in the data. When data transformation was not required, the non-transformed adjusted data and SEM is presented. When transformation was required, the back-transformed means and non-transformed SEM is provided, and the original transformation used on the data is specified. The transformations used (depending on the nature and skewedness the most relevant transformation was used for each variable) were square root (referred to throughout as Sqrt), logarithm base 10 (Log10) and Log 10(1 + x) (Log10, 1 + x). The Log 10 (1 + x) transformation has been used in cases where the data set contained zeros, for example in the case of fight number per hour, as a simple Log 10 transformation is not suitable for data sets which contain zeros, so all data was added to 1. All data were analysed using the Statistics Package for the Social Sciences (SPSS) v20.0 (IBM, Armonk, New York, NY, USA) using a general linear mixed model.

For sow data, pen was used as the experimental unit, with all measurements taken as individual sow results and then averaged across the pen. This was done to bring the data back to an average per sow, rather than total per 6 sows, to allow easier interpretation of the data. Replicate, day of measurement and treatment were fitted as fixed effects. Pen by day was fitted as a repeated effect. Parity group was originally in the model as a fixed effect, however, as we selected sows for even parity mix within groups, it did not significantly affect any measure and therefore, was removed from the model (parity 1–7, 3.7 ± 0.8). Reproductive performance was analysed with sows as the unit, changing the model so that sow by day was a repeated effect, and adding parity group (sows grouped into parity 1, parity 2–3 and parity 4+). For piglet data, the sow was the unit and a general linear model was used, with treatment, replicate and day included as a fixed effect. Litter size at selection (9.95 ± 1.4), sow parity (3.7 ± 0.8) and piglet age at weaning (27.04 ± 2.05) were used as covariates and sow by day as a repeated effect. The change in measurements from day to day was also analysed, by subtracting the earlier day from the latter.

## 3. Results

### 3.1. Injury

Total injuries and front injuries were greater in the SEP group on M0 and M1, than MS and WEAN (Table 2, *p* < 0.001). The MS group had fewer total injuries on day M1 and M6 than other treatments, fewer front injuries on M0 and M1 than MAI and SEP and fewer front injuries on M6 than all other treatments (Table 2). MAI sows also had greater front injury number than WEAN on M0 and M1 (Table 2). The number of fresh injuries was also affected by the day of mixing (*p* < 0.0001), but not treatment (*p* > 0.05) or mixing day by treatment (*p* > 0.05). A greater number of fresh injuries was observed on M0 (2.0 ± 0.3 back-transformed mean (LOG transformation) and non-transformed SEM) than on M-1 (0.02 ± 0.3), M1 (0.5 ± 0.3) and M6 (0.6 ± 0.3).

### 3.2. Aggressive Behaviour

Fight number per hour, overall percentage of time spent fighting and duration of individual fights (in seconds) were not affected by treatment by day (*p* > 0.05). There were no fights on M1 or M6 in the MS group (Table 2). When looking at the effect of day alone fight number (*p* < 0.0001), duration (*p* < 0.0001) and total percentage of observation time spent fighting (*p* < 0.05) were all higher on M0 compared to both other days (Table 3).

Treatment also had an effect on fight parameters. Number of fights per hour was lower in the MS group compared to the SEP group (*p* < 0.05, Table 4). The duration of individual fights was also affected by treatment, with lower duration in the MS treatment than all other treatments, with individual fights lasting over 10 s less per fight. Treatment affected the number of bites and knocks delivered, with more bites delivered when pooled across the days in the SEP group compared to all others and also fewer bites in the MS group compared to all others (*p* < 0.0001, Table 4).

There were more knocks by the SEP group than the MS group (*p* < 0.05, Table 4). More bites were also delivered on M0 than M1 and M6 (Table 3, *p* < 0.001) and more knocks were delivered on M0 and M6 than M1 (Table 3, *p* < 0.001). The number of displacements was affected by day, with more displacements on M0 than both M1 and M6 (Table 3, *p* < 0.05).

### 3.3. Other Behaviours

**Figure 3:**
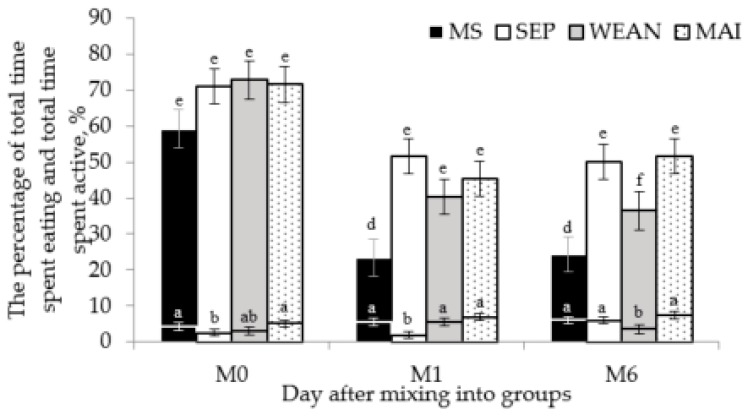
Percentage of time spent eating (lower bars) and active (upper bars) in the days after mixing ^1,2^. ^a,b,c,^ Means with different superscripts differ *(p* < 0.05). ^d,e,f^ Means with different superscripts differ (*p* < 0.05). ^1^ SEP: sow separation from crates and litters for seven hours daily (by mixing into a group pen and later returning to their crates and litters, MS; group-housed sows and litters (multisuckle) from day 21 lactation, WEAN; sows mixed after weaning at day 28 lactation, MAI; sows mixed after their post-weaning AI (4 ± 1 days after the last insemination). ^2^ Non-transformed and adjusted data are presented, the original transformation used for analysis of this data is the Sqrt transformation.

The percentage of total amount of time spent active was affected by the interaction of day by treatment (*p* < 0.05), with decreased activity in the MS sows compared to all other treatments on M1 and M6 and decreased activity in the WEAN group on M6 (Figure 3). There was also an effect of day on the percentage of time that sows spent eating (*p* < 0.05), with decreased time spent eating in SEP compared with the MS on M0 and M1 and decreased time spent eating in the WEAN group on M6 compared to all other treatments (Figure 3).

The percentage of time spent exploring around mixing was not affected by treatment, but there was an increased percentage of time spent exploring on M0 than on M1 (Table 3, *p* < 0.05). There was no effect of treatment on the number of mounting events, percentage of time spent mounting or duration of mounting events (*p* > 0.05).

### 3.4. Salivary Cortisol Concentration

Cortisol level was unaffected by treatment (*p* > 0.05: SEP; 34.27 ± 9.37 ng/mL, MS; 23.82 ± 9.69, MAI; 29.17 ± 9.69, WEAN; 32.93 ± 11.31) or M-day (*p* > 0.05: M-1; 20.98 ± 11.00 ng/mL, M0; 26.18 ± 10.09, M1; 28.89 ± 9.80, M6; 41.1 ± 9.48) or the interaction between the two (*p* > 0.05).

### 3.5. Reproduction

The MS and SEP treatments had fewer average days from weaning to first standing heat (*p* < 0.005) and a greater level of lactation oestrus (*p* < 0.0001) than the WEAN and MAI sows (Table 5). There was no difference in pregnancy rate in sows which were mated or in the total number of sows to not show oestrus among treatments (*p* > 0.05, Table 5). Treatments mixed after AI had no sows which did not cycle, and the SEP treatment had the most. MS sows had the highest pregnancy rate (Table 5). However, the total number of piglets born in the subsequent litter was affected by treatment, with an increased total number of piglets born in the MS treatment than in the MAI treatment (Table 5).

**Figure 4:**
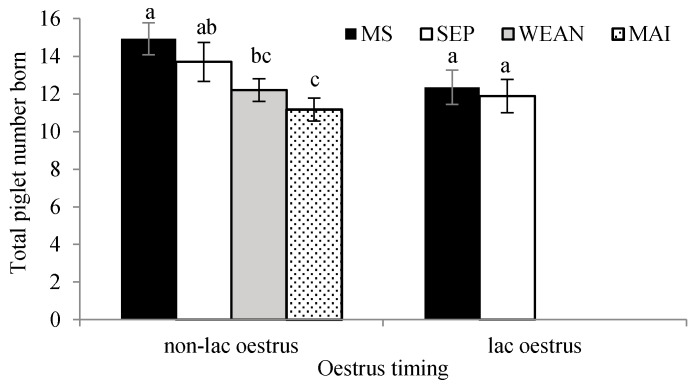
The total number of piglets born to sows which were mated in lactation or mated outside of lactation ^1,2^. ^a,b,c^ Means with different superscripts differ (*p* < 0.05). ^1^ Mean and SEM presented. ^2^ SEP: sow separation from crates and litters for seven hours daily (by mixing into a group pen and later returning to their crates and litters, MS; group-housed sows and litters (multisuckle) from day 21 lactation, WEAN; sows mixed after weaning at day 28 lactation, MAI; sows mixed after their post-weaning AI (4 ± 1 days after the last insemination). “lac”: lactational.

When analysed with sow as the unit and split into non-lactation oestrus (oestrus one day after the day of weaning and later) and lactation oestrus (on the day of after weaning or any time before), there were greater piglet numbers in subsequent litters in MS sows in the non-lactation oestrus group than both MAI and WEAN and also in the SEP group than WEAN (Figure 4).

The number of days between farrowing and oestrus were not affected when analysed with pen as the unit. With sow as the unit, there were fewer days between farrowing and oestrus in SEP (28.7 ± 0.7 days) compared to the MAI (31.5 ± 0.6 days) group (*p* < 0.005). The days from farrowing to oestrus were also affected by lactation oestrus, with lactation oestrus sows coming into oestrus 25.8 ± 0.6 days after farrowing, versus sows that did not come onto heat in lactation, which came into oestrus 31.4 ± 0.3 days after farrowing (*p* < 0.0001).

### 3.6. Piglet Results

#### 3.6.1. Piglet Weights

There was no treatment effect on the weight of piglets, or interaction between treatment and day (*p* > 0.05). There was a day effect, with increasing piglet weight with day (*p* < 0.001, Table 6).

When analysing weight change over time, there was a growth check at weaning, as seen by a significant difference between the days before weaning and after (Lac 20 to Lac 24 = 1.02 ± 0.15a kg, Lac 24 to Lac 28/W = 1.06 ± 0.15a kg, Lac28/W to W1 = 0.17 ± 0.15b kg and W1 to W2 = 0.06 ± 0.15b kg). Weight change was not significantly affected by the interaction between day and treatment (Table 7).

#### 3.6.2. Piglet Total Circulating Cortisol Concentrations

**Figure 5:**
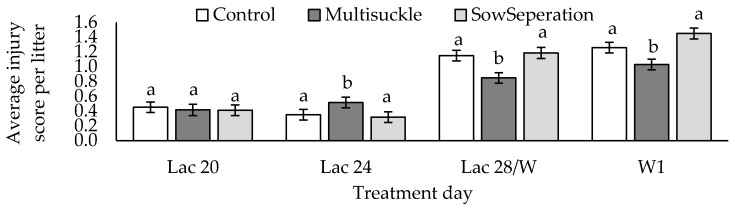
The effect of lactation housing methods on the injury score of piglets (Different superscripts note significant differences within treatment day, ^a,b^ *p* < 0.001, adjusted non-transformed mean and SEM are presented (data was Sqrt transformed for analysis).

There were significant effects of day on the circulating total cortisol concentrations of piglets from sows in different lactation housing conditions (*p* < 0.005). Cortisol is much higher on the day following weaning than in both lactation days measured (Table 6). Again, as with the piglet weights, treatment by day effect on total cortisol concentrations was not significant (*p* > 0.05, Figure 5). The change in cortisol was not significantly affected by any variable. However, Table 7 shows a decrease in cortisol in the MS group from Lac 24 to W1, compared to a large increase in the other two treatments, although this is not significant (Table 7, *p* > 0.05).

#### 3.6.3. Piglet Injury Scores

There were significant differences in piglet injury score for both day (*p* < 0.001) and day by treatment (*p* < 0.001). Piglet injury was significantly higher, following weaning, than when measured in lactation housing (Table 7). When looking at the effect of treatment by day, the piglets from multisuckle sows had significantly higher injury on Lac 24, following commencement of the lactation housing treatments, and significantly lower injury scores following weaning (*p* < 0.001, Figure 5).

Change in injury score was affected significantly by day by treatment (Table 7, *p* < 0.0005). Injuries increased more in the SEP piglets than in other treatments between Lac 20 and Lac 24. Injuries decreased in the MS groups and increased in both other treatments between Lac 24 and Lac28/W, also SEP injuries increased more than the C group from Lac28/W to W1, with the MS treatment as an intermediate.

#### 3.6.4. Piglet Mortality

There was one piglet death per treatment group for the duration of the trial, resulting in a mortality rate of 0.36% across all treatment groups.

## 4. Discussion

### 4.1. Mixing Day

Regardless of the effect of mixing time or lactation housing, it is clear that aggression was much greater on the day of mixing than the days following. This was seen in increased total, front and fresh injury, increased fight number, duration and percentage of total time spent fighting and increased displacements on the day of mixing. This is generally to be expected, as it is well known that aggression subsides as hierarchies form. This has been seen prior, in a study using animals from the same farm, which looked at space allowance at mixing and still found increased aggression on the day of mixing [9].

### 4.2. Sow Multisuckle Housing in Lactation

Examining aggression and injury in this cohort of sows, it is clear that the two different lactation-housing treatments imposed are almost polar opposites. The MS treatment results in lower levels of aggression and injury, the SEP group in higher levels, and the more commonly used MAI and WEAN treatments sitting in the middle. This can be seen with all measures of aggression and the levels of injury in each group. There was so little aggression in the MS group that there were no fights coded in the days following mixing. Due to the low animal numbers this is not a statistically significant difference to the number of fights per hour in the other groups. However, to have no fights at all in the days following mixing is an important observation.

MS sows were less active than the other treatments after mixing. This may have been caused by the HPA axis attenuation resulting in less anxious or restless animals in lactation [18]. Alternatively, the presence of piglets may minimize the movement of sows, whose maternal instincts minimize standing and laying behaviours to avoid crushing piglets [24,25]. Oxytocin release may also have played a part in reducing the aggression and activity seen in the MS group. The release of oxytocin has been linked to reduction in stress and anxiety and an increase in social activity [26]. This could mean that the circulating oxytocin in the multisuckle group could have decreased the sows stress but increased their social motivations, resulting in a more harmonious mixing event. It also seems that the stress of the SEP treatment, may have overridden these ‘relaxing’ influences, as the same effects are not seen in our SEP lactation mixing. However, due to the separation from piglets, perhaps stress levels were increased and their oxytocin circulation affected.

An important finding was that sows housed in a multisuckle system displayed improvements in reproduction in the subsequent litter (although the animal numbers may necessitate a larger trial under the same conditions to confirm this result). As was hypothesized, this could be attributed to the amelioration of aggression (and thus stress) during follicular growth and implantation [10,17]. Perhaps an additional explanation is the more natural weaning process that occurs in multisuckle systems [5]. During lactation luteinizing hormone (LH) secretion is suppressed, due to endogenous opiates that are released due to suckling, which suppress the release of GnRH [27]. Therefore, the more natural weaning process in the MS group may have led to a decrease in suckling suppression on LH and therefore increased follicle growth in lactation, more ovulations and better-quality oocytes. This has also been reported by Terry et al. (2013), who found that with four treatments; control and three split wean treatments (with three, five or seven of the heaviest piglets removed from the sow on Day 18 lactation), the control sows had a lower subsequent total born [28]. One disadvantage of multisuckle systems is that they can result in increases in pre-weaning mortality (Thomsson et al., 2016) [29]. If this is in fact the case, our results suggest that this could be offset by better subsequent reproduction with multisuckle systems.

One trial design element that may have affected sow behaviour, and therefore, the levels of aggression in MS sows, was the greater space allowance accompanying this housing method. This was unavoidable, but the trial design ensured that each MS sow and litter had the same footprint as in a crate. Space allowance may contribute to decreasing the aggression in the MS sows, however, the space provided for treatments is that which is likely to be the industry norm for implementation of each housing method. A recent paper by Greenwood et al. (2016) in the same shed system with the same genetics (with greater animal numbers) compared 2, 4 and 6 m^2^/sow and demonstrated significant differences, with increased space equaling decreased aggression [9]. However, the differences seen with these space allowances were minimal compared to the effects of the multisuckle housing in this current study.

### 4.3. Sow Socialisation and Separation from Piglets in Lactation

The SEP treatment group recorded the highest levels of aggression over all treatments (through fight, bite and knock number) and high injury number. Injuries in the SEP group increase sharply and then plateau, suggesting that injuries in this group were more severe on the day of mixing and took a long time to heal, but that after mixing day fewer injuries were sustained. This concurs with the behaviour in this group, with more fights per hour on M0 and M1 than any other treatment group. Cortisol was not affected by the treatments. Cortisol synthesis differs greatly in different stages of the cycle and between different sows. It could be that larger animal numbers were needed to see a significance, or just simply that the treatments had no effect on the cortisol level of the sows. WEAN and MAI sows did have longer fight duration than the SEP sows, but fewer fights, bites, knocks and injury, suggesting that the fights were longer, but less intense than SEP. Overall this suggests higher intensity fighting in SEP on M0 and M1 with correspondingly increased injuries. Another explanation for the greater number of injuries with the implementation of the piglet separation treatment, is that the injuries may have been caused by suckling piglets, who, following separation from the sow were noted to suckle ferociously upon return (anecdotally) and it is likely that they caused injury through this.

Following mixing, SEP sows spent less time eating. This may be due to increased competition for food and was unexpected, since the SEP and MS sows were fed ad libitum at the time of mixing, which should have resulted in longer eating duration and less feed competition. It is possible that sows learnt that they would be returning to their crates and would receive their evening ration in individual feeders, resulting in decreased motivation to defend the resource in the morning feed. However, the decreased time spent eating lines up with the days of higher aggression in this group. Anecdotally, there appeared to be decreased ad libitum feed amount needed in this group versus the multisuckle group (in order to avoid feed wastage), although this was not officially recorded. This is a concern since the treatment would appear to be affecting their nutrition. However, decreased time spent with the litter may result in a decreased nutritional requirement for the sow.

Although SEP sows were separated from piglets for 7 h daily, and MS sows remained with their litters, there was no difference between the numbers of sows expressing oestrus or lactational oestrus. Considering both lactation and post-weaning oestrus together, MS and SEP sows came into oestrus, on average, one day after weaning. This shows that the difference in suckling stimuli between the two groups mixed in lactation did not alter oestrus expression. Previous research suggests that the frequency of nursing is decreased overall and declines more rapidly after introduction of sows into ‘getaway’ systems [30] (our SEP treatment involved imposed separation whilst ‘getaway’ systems allow choice). There are a few possible explanations for why our oestrus onset was not affected. Nursing might not have decreased as expected in the separation group as, due to the enforced separation, suckling frequency when the sow was present may have increased. Another explanation could be that suckling decrease was similar in both treatments, with a gradual and natural decrease in the MS group, and a forced 7 h decrease in SEP. Alternatively, irrespective of the treatment imposed on the sows, perhaps no more animals would have been able to achieve lactation oestrus, due to factors such as their genetics, natural cycles and condition after lactation.

### 4.4. Mixing Sows After Weaning

An important question posed by these treatments is whether it is best to mix before or after insemination when regarding the aggression and resulting injury, as it is common for these two methods to be used in the Australian industry. MAI sows had more front injuries on M0 and M1 than WEAN, suggesting more reciprocal fighting (although fight number and duration were not affected). However, the wean group spent less time eating and less time active on M6 then the MAI group and these results may indicate higher levels of stress in the WEAN group even 6 days after mixing. This was also reported by Rault et al. (2014), with increased plasma cortisol concentrations on d1 following mixing and increased weight loss in sows mixed at weaning, compared to those mixed after AI [31]. These behavioural differences are more likely to be caused by sows coming into oestrus (around six days following weaning) or the decrease of ad libitum feed level to 2.5 kg at this time. It may also be due to the ‘double-stress’ of weaning and mixing at the same time, prolonging the formation of hierarchy. The increased time to settle in WEAN and increased front injuries in MAI make it difficult to conclude which of these methods is optimal.

The increased adoption of group housing has created interest into when the best time to group sows is relative to insemination. We discussed that if done before AI, this could have less of an impact on reproductive outcomes as the aggression should have subsided before AI. However, Rault et al. (2014) showed that such a strategy could influence sexual receptivity [31]. We hypothesized that the stress of weaning coupled with oestrus and grouping may act to increase sow aggression when compared to those that were grouped after AI (5–10 days after weaning). Given the small number of sows used in this experiment, we could not determine any differences in reproductive outcomes between the two. However, the WEAN group had a percentage of sows that did not exhibit oestrus, while the MAI group had all of its sows exhibit oestrus, although there was no significant difference between the groups.

The timing of oestrus was not affected by housing type after weaning, with oestrus on average 4 days after weaning in both WEAN and MIA treatments. Although not significant, the MAI sows had 10%–13% decrease in conception, when compared to the other treatments, and it is likely that this difference would have been significant with greater numbers. These results contrast with previous trials, which reported delayed oestrus, variation in the onset of oestrus and decreased mating success, when sows were housed in groups from weaning compared to after AI [27,31]. For this reason, it has been suggested that oestrus detection protocols are especially important in group-weaned systems [31,32].

### 4.5. The Piglets

#### 4.5.1. Mortality in Alternative Lactation Systems

In the current study, the incidence of piglet mortality was low, with only one piglet dying in each of the Control and Multisuckle systems, equating to a mortality rate of 0.36% in all treatment groups. Due to most mortalities occurring within the first week after birth, little documentation of mortalities in these alternative lactation systems is available. An on-farm study comparing multisuckling to farrowing crate housing, conducted by Hulten et al. (1997), revealed that from piglets 21 days to 42 days had a mortality rate of 6.5% vs 1.4%, respectively, during the multisuckling phase [33]. In smaller scale studies, there has been no significance found in mortality between systems during the multisuckling phase (14–21 until 28 days) [34,35]. Therefore, a mortality rate of 0.36% during the multisuckle phase argues against the view that improved sow welfare and mobility coincides with high mortalities, suggesting that multisuckle systems may be a viable option, at least later in lactation.

Lactation length has been reduced over the years to increase the number of litters a sow may produce per year. This reduction in lactation length results in earlier piglet weaning (21 to 28 days) and a more drastic response shown by the piglets to weaning, consequently an effect of stress and poor welfare [36]. Thomsson et al. (2016) looked at the effects of imposing a multisuckle treatment at one, two and three weeks after weaning and found that overall piglet mortality was positively correlated with mortality in the multi-suckling pen for piglets group housed at one week and at two weeks post-farrowing, but not for piglets group housed at three weeks post-farrowing [29]. This particular research shows us that overall piglet mortality is not affected in multisuckle groups if sows and piglets are group housed at three weeks post-farrowing. Verdon et al. (2017) had similar results, with decreasing mortality with increasing piglet age at the implementation of multisuckle housing. They found that piglets mixed at 7 days of age had the highest mortality (17%) over those mixed at 10 days of age (12%) and those at 14 days of age (8%) [37]. These results might explain why we had such low mortality rates, as we mixed into multisuckle housing on day 21 of lactation.

#### 4.5.2. Piglet Growth and Final Weights

Research conducted by Lambertz et al. (2015) revealed that loose housed and farrowing crate litters did not differ in weight on days, 1, 7, 14 and 26 post-partum [38]. This is in line with our results, as the weight of MS and C piglets are similar for the duration of the experimental period. The SEP piglets appear to have had a weight response to weaning, which was not seen to the same extent in the other two treatments.

Previous studies suggest that group housing pre-weaning results in a reduced or absent post-weaning growth check and consequently, the improved growth rate of multisuckling treatment during the weaning period [5]. Factors contributing to this better adjustment at weaning are improved social skills, the absence of mixing at weaning (already mixed partially) and increased experience with creep feed [39]. Hessel et al. (2006) [40] and Kutzer et al. (2009) [41] indicated that in the five weeks post-weaning, piglets that were housed in systems allowing piglet cross contact, such as multisuckling, weighed 0.8–1.0 kg more pre-weaning.

The sow separation treatment endeavored to reduce the effects of the growth check by stimulating the consumption of creep feed, due to the isolation of the sow and inability to suckle, better adjusting the piglet to this isolation in an attempt to replicate weaning. Berkeveld et al. (2009) using 12 h isolation [39] and Kuller et al. (2004) using 7 h isolation [42] were able to replicate such a response. Kuller et al. (2004) indicated that periods of short fasting (7 h isolation) has a stimulating effect on post-weaning feed intake despite body weight at weaning being reduced, promoting improved growth across the weaning period, contradictory to our findings [42]. However, dry feed intake (creep feed) pre-weaning was concluded by Thymann et al. (2007) to not be the major contributing factor to improved growth around weaning but did contribute to an improved and delayed response to the stressors of weaning [43]. It could be that the SEP piglets have been programmed to expect the return of the sow, and therefore, have less motivation to compete for a space at the feeder.

#### 4.5.3. Piglet Stress and Injury

The current data indicate that piglets housed in multisuckle accommodation had the lowest incidence of injury scores at weaning and the lowest cortisol response to weaning, but this is not significant. Cortisol is known to rise in response to both positive and negative states in swine [44]. Therefore, a combination of injury scoring and cortisol levels is needed to establish whether the response seen in free plasma cortisol concentrations is a response to either excitement or stress. Multisuckling offers the ability to adapt to solid feed through exploratory behaviour without the additional stress of sow isolation, a response of curiosity rather than necessity [45,46]. Improved socialization pre-weaning is known to improve welfare and in some cases performance in piglets post-weaning [3,40]. The reduced cortisol and injury at weaning and mortality results show us that multisuckle housing in late gestation may improve piglet welfare at no cost to production. We must take into consideration, that there is also a rise in injury (not mirrored this time with cortisol) when the multisuckle treatment is imposed. It is still difficult to decide if, for the piglet, the move to multisuckle is just displacing the stress rather than reducing overall stress levels. A measure that would have improved the science of this paper is the future growth rate of the piglets, to see it if the SEP piglets caught up and if the MS piglets stayed in line with the C piglets (traditionally weaned WEAN and MAI). Recent work by Verdon et al. (2016) has filled some of the gaps in this area, finding that piglets housed in groups in lactation deliver fewer bouts of aggression, fight less frequently in the 2 h post-mixing at weaning and sustain fewer skin injuries 24 h later, than piglets housed in crated or pig safe pens [47]. However, the research into this area is inconclusive as to if the growth check in lactation, which comes with the implementation of multisuckle treatments, is worth it to reduce the growth check at weaning, and what later effects these different stressors have on growth and production.

## 5. Conclusions

We were able to show conclusively that mixing sows during lactation, in the presence of their piglets, reduced aggression and injuries, and improved subsequent reproduction with no apparent ill effects on their piglets. The mixing of sows during lactation, while separated from piglets, increased aggression levels. Based on the current data, it is not possible to determine which of the more commonly used sow mixing times, at weaning or after insemination, should be recommended to minimize the effects of mixing. Due to the substantial differences between the results from the MS and SEP treatments, we cannot support or dispute the hypothesis that mixing prior to insemination is a more economical practice, therefore, further work is required in this area. Due to the substantial differences between the results from the MS and SEP treatments, further work is required to pick apart the effects of mixing in lactation.

## Figures and Tables

**Table 1:** Ethogram of behaviours with added behaviours for specific use in multisuckle (MS) housing analysis, with the presence of piglets, and for coding of boar exposure and defined behaviour type used to analyse the behaviour.

Active, resting, eating and drinking	Sows were classed as eating or drinking if they were obviously doing either of the two. If the sow’s behaviour was unclear, she was coded as active. If a sow was dog sitting, standing or walking she was considered active. Sows were considered resting if flat to the floor.	Continuous events
Exploring	Actively manipulating and exploring the surrounding environment, such as rooting, moving drinkers and chewing fences.	Continuous event
Displacement	Movement of one sow by another, from a valued resource such as food, drinker or lying space (if multiple knocks or bites are required, this is a fight).	Point event
Fighting	Aggression lasting >3 s, including two or more knocks or bites. Aggression can be reciprocal or non-reciprocal.	Continuous event
Knock	One sow knocks another sow using her head and neck, contacting any part of the receiving sow.	Point event
Bite	One single bite delivered from one sow to any part of another.	Point event
Lunge	Sow lunges at another but does not make physical contact	Point event
Flee	Sow moves herself quickly and as far away as she can get from another sow, in response to an aggressive action.	Point event
Mounting	One sow mounts another, with her front legs both over the back of the other animal.	Point event
Suckling	Sows in multisuckle housing could be coded as suckling: Suckling began when sows began to actively grunt on their side and piglets rushed to the udder. Suckling finished when sows rolled onto their udder, blocking access to the piglets.	Continuous
Interaction with piglets (non-aggressive or aggressive)	Exclusive to sows in multisuckle housing. Investigation of piglets, including nose-to-nose contact and moving the piglets around. The modifier aggressive or non-aggressive was used. Interaction was considered as aggressive if piglets were pushed with force so that they fell, were thrown, or were bitten.	Continuous
Boar exposure	Not coded for MAI. Boar exposure was coded from the start of exposure, to when the boar was no longer present or the sows returned to their housing. There were no other behaviours coded while boar exposure was coded for, leaving a blank section of video when exposure was taking place.	Continuous
Total time active	All continuous behaviour codes considered as a sow being active were summed, allowing for differences in behaviour between treatments, such as suckling in the MS treatment group (all behaviour except time spent resting).	Calculated after

**Table 2:** Interaction between days after mixing and treatment on measures linked to aggression in sows ^1,2^.

Mixing Day	Treatment	Total Injury, Number per Sow	Front Total Injury, Number	Fights per Hour, Number
M-1	MS	5.6 ± 4.2 ^a^	2.0 ± 2.1 ^a^	
	SEP	6.7 ± 4.2 ^a^	2.5 ± 2.1 ^a^	
	WEAN	4.4 ± 3.8 ^a^	1.8 ± 1.9 ^a^	
	MAI	4.4 ± 3.8 ^a^	2.1 ± 1.9 ^a^	
M0	MS	14.6 ± 3.8 ^a^	6.7 ± 1.9 ^a^	0.27 ± 0.07
	SEP	32.3 ± 3.8 ^b^	15.1 ± 1.9 ^b^	0.48 ± 0.07
	WEAN	16.3 ± 3.8 ^a^	7.2 ± 1.9 ^a^	0.27 ± 0.07
	MAI	19.7 ± 3.8 ^a^	11.2 ± 1.9 ^b^	0.20 ± 0.07
M1	MS	11.4 ± 4.2 ^a^	5.1 ± 2.1 ^a^	0.00 + 0.06
	SEP	34.4 ± 3.8 ^b^	15.9 ± 1.9 ^b^	0.10 ± 0.06
	WEAN	20.7 ± 3.8 ^c^	10.8 ± 1.9 ^a^	0.06 ± 0.06
	MAI	21.6 ± 3.8 ^c^	14.3 ± 1.9 ^b^	0.06 ± 0.06
M6	MS	9.5 ± 3.8 ^a^	4.1 ± 1.9 ^a^	0.00 ± 0.06
	SEP	36.1 ± 3.8 ^b^	18.2 ± 1.9 ^b^	0.08 ± 0.06
	WEAN	39.1 ± 3.8 ^b^	20.3 ± 1.9 ^b^	0.13 ± 0.07
	MAI	40.9 ± 3.8 ^b^	24.0 ± 1.9 ^b^	0.06 ± 0.06
Original Transformation		Sqrt	Sqrt	Lg10(1 + x)
*p* value		<0.0001	<0.0001	>0.05

^a,b,c^ Means within a column and day, with different superscripts differ (*p* < 0.05). ^1^ Non-transformed and adjusted means ± SEM are presented when transformations were needed and the original transformation is specified in the table. Transformation abbreviations are square root (Sqrt), logarithm base 10 (Lg10) and Log 10 (1 + x) (Lg10, 1 + x). ^2^ SEP: sow separation from crates and litters for seven hours daily (by mixing into a group pen and later returning to their crates and litters), MS: group-housed sows and litters (multisuckle) from day 21 lactation, WEAN: sows mixed after weaning at day 28 lactation, MAI: sows mixed after their post-weaning AI (4 ± 1 days after the last insemination).

**Table 3:** The effect of days after mixing on behavioural measures in group housed sows mixed at different times ^1^.

Behaviour	M0	M1	M6	Trans.	*p* Value
fights per hour	0.3 ± 0.03 ^a^	0.05 ± 0.03 ^b^	0.07 ± 0.03 ^b^	Lg^10^_(_1 + x)	<0.0001
fight duration, second	34.1 ± 6.2 ^a^	3.1 ± 6.0 ^b^	3.9 ± 6.0 ^b^	Lg^10^_(_1 + x)	<0.0001
Time spent fighting, %	0.3 ± 0.08	0.004 ± 0.08	0.08 ± 0.08	NA	<0.05
bites per sow per hr	2.4 ± 0.2 ^a^	0.3 ± 0.2 ^b^	0.6 ± 0.02 ^b^	Lg^10^(1 + x)	0.001
knocks per sow per hr	1.2 ± 0.3 ^a^	0.4 ± 0.2 ^b^	1.2 ± 0.2 ^a^	Lg^10^(1 + x)	< 0.001
displacements, per sow per hr	0.4 ± 0.04 ^a^	0.1 ± 0.04 ^b^	0.2 ± 0.04 ^b^	Log	<0.05
spent exploring, %	2.3 ± 0.4 ^a^	1.4 ± 0.4 ^b^	2.2 ± 0.1 ^ab^	Sqrt	<0.05

^a,b^ Means within a row with different superscripts differ (*p* < 0.05). ^1^ Non-transformed and adjusted means ± SEM are presented with the transformation used for the statistical analysis provided in the table for reference (Trans. Column). Transformation abbreviations are square root (Sqrt), logarithm base 10 (Lg10) and Log 10 (1 + x) (Lg10, 1 + x), and not applicable or no transformation (NA).

**Table 4:** Effect of treatment on gestating sows on measures of aggression delivered per hour in the days after mixing ^1,2^.

Behaviour	MS	SEP	WEAN	MAI	Trans.	*p* Value
fights per hour, per sow	0.08 ± 0.04 ^a^	0.20 ± 0.03 ^b^	0.15 ± 0.04 ^ab^	0.11 ± 0.03 ^ab^	Lg^10^(1 + x)	<0.05
fight duration, seconds	3.0 ± 7.5 ^a^	15.6 ± 7.0 ^b^	16.9 ± 7.5 ^b^	16.5 ± 7.0 ^b^	Lg^10^(1 + x)	<0.05
Bites per hour, per sow	0.4 ± 0.2 ^a^	2.1 ± 0.2 ^b^	1.0 ± 0.2 ^c^	0.8 ± 0.2 ^c^	Lg^10^(1 + x)	<0.0001
Knocks per hour, per sow	0.4 ± 0.3 ^a^	1.5 ± 0.3 ^b^	1.1 ± 0.3 ^ab^	0.7 ± 0.3 ^ab^	Lg10	<0.05

^a,b^ Means within a row with different superscripts differ (*p* < 0.05). ^1^ Non-transformed adjusted means ± SEM are presented with the transformation used for the statistical analysis provided in the table for reference (Trans. Column). Transformation abbreviations from analysis are logarithm base 10 (Lg10) and Log 10 (1 + x) (Lg10, 1 + x). ^2^ SEP: sow separation from crates and litters for seven hours daily (by mixing into a group pen and later returning to their crates and litters), MS: group-housed sows and litters (multisuckle) from day 21 lactation, WEAN: sows mixed after weaning at day 28 lactation, MAI: sows mixed after their post-weaning AI (4 ± 1 days after the last insemination).

**Table 5:** Effect of treatment on reproduction in different housing systems from lactation in sows ^1,2^.

Behaviour	MS	SEP	WEAN	MAI	Trans.	*p* Value
Sows which failed to show oestrus, %	6.7 ± 4.9	16.7 ± 4.9	10.0 ± 4.9	0.0 ± 4.9	NA	>0.1
Pregnant, % sows mated	93.3 ± 7.0	90.0 ± 7.0	91.7 ± 7.0	80.0 ± 7.0	NA	>0.05
Total born, piglets	13.8 ± 0.5 ^a^	12.2 ± 0.5 ^a,b^	12.3 ± 0.5 ^a,b^	11.0 ± 0.5 ^b^	Sqrt	<0.05
Number of days from weaning to oestrus	1.0 ± 0.7 ^a^	1.0 ± 0.7 ^a^	4.3 ± 0.7 ^b^	4.9 ± 0.7 ^b^	NA	<0.005
Lactation oestrus, %	50.7 ± 10.1 ^a^	61.7 ± 10.1 ^a^	0.0 ± 10.1 ^b^	0.0 ± 10.1 ^b^	Sqrt	<0.0001

^a,b^ Means within a row with different superscripts differ (*p* < 0.05). ^1^ Non-transformed and adjusted means ± SEM are presented with transformations use for statistical analysis specified in the Trans. column. Transformation abbreviations are: square root (Sqrt), logarithm base 10 (Lg10) and Log 10 (1 + x) (Lg10, 1 + x). ^2^ SEP: sow separation from crates and litters for seven hours daily (by mixing into a group pen and later returning to their crates and litters, MS; group-housed sows and litters (multisuckle) from day 21 lactation, WEAN; sows mixed after weaning at day 28 lactation, MAI; sows mixed after their post-weaning AI (4 ± 1 days after the last insemination).

**Table 6:** The effect of days around weaning on piglet condition and stress.

Heading	Baseline (Lac 19/20)	Lac 24 ^1^	Lac 28/W ^1^	W1 ^1^	W2 ^1^	Tran.	*p* Value
Weight, kg	* 6.1 ± 0.2 ^a^	7.2 ± 0.2 ^b^	8.1 ± 0.2 ^c^	8.3 ± 0.2 ^c^	8.3 ± 0.2 ^c^	NA	<0.001
Injury score	* 0.43 ± 0.04 ^a^	0.39 ± 0.04 ^a^	1.06 ± 0.04 ^b^	1.25 ± 0.04 ^b^		Sqrt	<0.001
Cortisol, ng/ml	^#^ 31.8 ± 4.6 ^a^	29.1 ± 4.4 ^a^		50.4 ± 4.6 ^b^		Lg^10^	<0.005

* Baseline sample taken on lactation day 20. ^#^ Baseline measure taken on lactation day 19. ^a,b,c^ Means with different superscripts differ across rows (*p* < 0.05) ^1^ The table headings refer to the day of lactation on which the measures were taken; the baseline day is before any lactation housing was altered and was either day 20 of lactation or day 19 depending on the measurement taken, Lac 24 is day 24 of lactation and follows implementation of the lactation housing treatments (on day 21) and is before the piglets were weaned, Lac 28/W is the last day of lactation, day 28 lactation, and the day on which the piglets were weaned, W1 is the day after the piglets were weaned and W2 is two days after the piglets were weaned.

**Table 7:** The change in piglet condition and stress and the effect of treatment by day.

Mixing Day ^2^	Treatment ^1^	Weight Change, kg	Injury Score Change	Mixing Day ^2^	Cortisol Change, ng/ml
Lac 20 to Lac 24	C	1.22 ± 0.26	−0.1 ± 0.08 ^a^	Lac 19 to Lac 24	−2.06 ± 10.27
	MS	0.8 ± 0.27	0.12 ± 0.09 ^b^		9.59 ± 14.25
	SEP	1.06 ± 0.27	−0.09 ± 0.09 ^a^		4.53 ± 11.77
Lac 24 to Lac 28/W	C	0.97 ± 0.26	0.77 ± 0.08 ^a^	Lac 24 to W1	29.34 ± 11.04
	MS	1.19 ± 0.27	0.31 ± 0.08 ^b^		−6.3 ± 12.95
	SEP	1.01 ± 0.27	0.87 ± 0.08 ^a^		21.95 ± 11.77
Lac 28/W to W1	C	0.03 ± 0.26	0.01 ± 0.08 ^a^		
	MS	0.09 ± 0.27	0.19 ± 0.09 ^ab^		
	SEP	0.39 ± 0.27	0.26 ± 0.09 ^b^		
W1 to W2	C	0.09 ± 0.26			
	MS	0.16 ± 0.27			
	SEP	-0.08 ± 0.27			
*p* value		>0.05	<0.0005		>0.05

^a,b^ Means with different superscripts differ within a column and day (*p* < 0.05) ^1^ C = control piglets, kept with sows in a home crate from birth until weaning at day 28 lactation, MS = Sows and piglets maintained in a crate until day 21 lactation and then mixed into a multisuckle group of 6 sows and their litters until weaning at day 28 lactation, SEP = Sows and piglets maintained in crates until day 21, after which point sows were removed from the crate for 7 h a day until weaning at day 28 lactation. ^2^ Abbreviations refer to the day of lactation on which the measures were taken; the baseline day is before any lactation housing was altered and was either day 20 of lactation or day 19 depending on the measurement taken (which is why cortisol change is measured from day 19). Lac refers to the day of lactation, for example Lac 24 is day 24 of lactation. Lac 28/W is the last day of lactation, day 28 lactation, and the day on which the piglets were weaned, W1 is the day after the piglets were weaned and W2 is two days after the piglets were weaned.
